# A Machine Learning Framework Integrating DeepLabCut and SimBA for Quantifying Aggressive Behavior in Swimming Crab *Portunus trituberculatus*

**DOI:** 10.3390/ani16101555

**Published:** 2026-05-20

**Authors:** Chuanlong Ding, Yuanyuan Fu, Zhiqiang Zhou, Ting Chen, Wuqiang Xia, Weijiang Huang, Bincai Zhou, Jiameng Chen, Cheng Zheng, Chunlin Wang, Changkao Mu, Changjun Liu, Lei Liu

**Affiliations:** 1 School of Marine Sciences, Ningbo University, Ningbo 315832, China; 13870316091@163.com (C.D.);; 2 Ningbo Institute of Oceanography, Ningbo 315832, China; 3 Aquatic Technology Promotion Station of Xiangshan County, Ningbo 315709, China; 4 Experimental Base of Xiangshan (Ningbo University) Aquatic Seed Industry Innovation Research Institute, Ningbo 315709, China

**Keywords:** *Portunus trituberculatus*, aggressive behavior, DeepLabCut, SimBA, behavioral quantification

## Abstract

The swimming crab *Portunus trituberculatus* is a commercially important species in aquaculture, and its aggressiveness strongly influences productivity, making quantitative assessment a priority. However, such an assessment is hindered by behavioral complexity and a lack of high-throughput approaches. Here, we present a machine learning framework for quantifying aggressive behavior using DeepLabCut-SIMBA (Simple Behavioral Analysis). By converting video data into quantifiable durations of aggression-related events, we established a Time-weighted Aggression Index (TAI) that enables standardized, high-throughput assessment. The TAI showed strong concordance with conventional methods, confirming its validity. To demonstrate its applicability, we examined the correlation between TAI and serotonin levels, revealing distinct physiology-behavior associations. We then stratified crabs into three aggression categories based on TAI and conducted pairwise fighting trials. The frequency of aggressive interactions differed significantly across group combinations, indicating that TAI-based grouping effectively predicts behavioral outcomes. This framework offers a reliable, scalable tool for behavioral phenotyping and practical value for selective breeding programs.

## Simple Summary

Swimming crabs are an important species for seafood farming, but they often fight with each other. These fights can cause injuries and reduce the number of healthy crabs, which is a problem for aquaculture producers. To better understand and manage crab fighting, scientists need a way to measure aggression that is fast, consistent, and not based on personal judgment. In this study, we created a computer program that watches videos of crabs and automatically detects when they are fighting. The program tracks the crabs’ movements and calculates a score. We call this score the Time-weighted Aggression Index. It tells us how aggressive a crab is. We tested this score and found that it matches well with traditional methods of measuring aggression. We also showed that crabs with higher scores actually fight more often when placed together. This automatic tool saves time and provides a reliable way to study crab aggression. It can help crab farmers reduce fighting in their tanks and could even be used in breeding programs to select less aggressive crabs. In the future, the same approach could be adapted to study fighting in other animals as well.

## 1. Introduction

In nature, the phenomenon of distinct personality traits in individual animals is widely observed [1,2]. Animal personality refers to a set of consistent behavioral characteristics exhibited by individual animals, as well as stable behavioral differences between individuals across time and/or contexts [1,3]. Explorative behaviour and risk-related traits such as boldness and aggressiveness are common characteristics of animal personalities [4,5]. In the current study, we specifically focus on aggressiveness, which can be defined as the propensity of animals to display aggressive behaviors toward conspecifics, with significant variation in aggressive tendencies observed among different individuals [6]. Notably, this behavioral trait is fundamentally linked to critical aspects of animal life history, including its capacity to acquire food resources, compete for mates, and establish dominant positions within social hierarchies [7,8]. The study of aggressive behavior has historically relied on manual observation—a method constrained by subjectivity and limited throughput. As Pereira et al. [9] review, recent advances in deep learning-based motion tracking and behavior classification are overcoming these limitations, providing automated, high-resolution quantification that was previously unimaginable. Choi and Kumar [10] describe how computational ethology is ushering in a new era for behavioral quantification, shifting the field from labor-intensive manual observation toward automated, high-resolution analysis across diverse experimental contexts. These methodological innovations open the door to quantifying individual variation in aggressiveness in ways not previously possible. Aggressive behavior represents a fundamental aspect of animal social interactions, serving critical functions in resource competition and territorial defense [11]. This is particularly evident in decapod crustaceans, especially the commercially important swimming crab *P. trituberculatus*, where agonistic behaviors significantly impact aquaculture productivity through cannibalism and injury-related losses [12]. Field observations have confirmed that cannibalism constitutes a primary cause of mortality and yield reduction in commercial crab farming operations [13]. Most recently, studies have demonstrated that frequent antagonistic interactions lead to substantial energy expenditure, elevated stress levels, limb loss, cannibalism, and reduced market value in crab species [14,15]. Given the considerable economic losses attributed to aggressive behaviors in crustacean aquaculture, accurately quantifying individual variation in aggressiveness is essential for informing selective breeding programs and optimizing culture practices. However, traditional behavioral assessment methods relying on manual observation suffer from inherent limitations, including subjective bias, low throughput, and poor inter-rater reliability [16,17]. Therefore, the development of robust quantitative approaches for aggression measurement represents a crucial step toward understanding crustacean behavioral ecology and improving husbandry practices [18].

In addition, the complex and ubiquitous expression of aggressive phenotypes in crustaceans poses challenges that increasingly exceed the capacity of conventional methodologies. Agonistic behaviors are commonly observed in many decapod crustaceans, including freshwater crayfish (*Astacus leptodactylus*), mud crab (*Scylla serrata*) [19], swimming crab [20], and Chinese mitten crabs (*Eriocheir sinensis*) [21]. The intensity of these agonistic interactions and the expression of aggressive behaviors are influenced by multiple factors, such as sex [22], body size differences [23,24], reproductive status [25], and individual aggressiveness. Individuals with varying levels of aggressiveness exhibit significant differences in fighting ability, suggesting that aggressive behavior is consistent within individuals [26]. Aggressiveness, as a personality trait, plays a crucial role in determining the combat performance of swimming crabs. Furthermore, neurotransmitters such as serotonin (5-HT) and dopamine (DA) are widely distributed in the central nervous system and play significant roles in physiological activities and behavioral states across many animal species, and are particularly recognized as key regulators of crustacean agonistic behaviors [27,28]. Existing research demonstrates that elevated 5-HT concentrations are associated with aggression promotion [29]. Specifically, 5-HT enhances attack motivation in paired *Astacus* crayfish while reducing retreat likelihood and prolonging fight duration [30].

Despite these advances, large-scale, high-precision quantitative investigations into the relationship between aggressiveness and neurotransmitter dynamics in crustaceans remain conspicuously absent. Given that aggressiveness has profound implications for individual fitness and aquaculture productivity, the development of appropriate quantitative methodologies is essential for elucidating the complex neurochemical mechanisms that govern agonistic behaviors. The application of such high-throughput, high-precision analytical approaches would not only advance fundamental understanding of behavioral regulation but also hold substantial practical value. In particular, the establishment of robust quantitative frameworks could facilitate selective breeding strategies aimed at mitigating the adverse effects of aggressive interactions, including cannibalism and injury-related losses, within aquaculture production systems.

Recent advances in computational ethology have revolutionized animal behavior research through deep learning-based methodologies that overcome critical limitations of conventional manual annotation by providing standardized, high-throughput behavioral quantification [18,31]. DeepLabCut, a state-of-the-art pose estimation framework, enables precise tracking of anatomical keypoints using deep neural networks [32]. When integrated with SimBA (Simple Behavioral Analysis), an accessible platform for supervised machine learning classification, researchers gain a comprehensive pipeline spanning from motion capture to behavioral phenotyping [33,34,35]. However, although these tools have found widespread application across neuroscience and behavioral ecology, their potential for crustacean aggression studies remains largely unexplored, as most existing studies focus on vertebrate models [36,37] and therefore lack specialized analytical frameworks for arthropod-specific behavioral repertoires. Furthermore, current aggression metrics often rely on oversimplified behavioral indices [38], failing to capture the multidimensional nature of agonistic interactions. Our study addresses these limitations by developing an integrated approach integrating both pose estimation and behavioral classification for aggression quantification in swimming crabs. While recent studies have demonstrated the feasibility of DeepLabCut and SimBA pipelines in other crustacean species [35], the challenge of capturing the continuous, graded nature of agonistic behavior remains unresolved. To address this gap, we introduce the Time-weighted Aggression Index (TAI), a continuous measure that integrates both the frequency, intensity, and duration of aggressive acts into a single score. The mathematical formula and calculation details are provided in the Results section. This index is embedded within a species-specific analytical framework that includes customized ethograms for *P. trituberculatus*, enabling automated, high-throughput assessment of individual variation in aggressiveness. This methodological innovation aims to advance fundamental research on aggressive behavior in crustaceans and provide practical tools for aquaculture management and selective breeding programs.

## 2. Materials and Methods

### 2.1. Animal Collection and Maintenance

The experiment was carried out at the Ningbo Institute of Oceanography in Zhejiang Province from 10 April to 29 June 2025. The experimental subjects, swimming crabs, were wild-caught from the waters near Xixuan Island. Only healthy male individuals with intact carapaces and limbs, weighing 60 ± 20 g, were selected for the experiment. Crabs that were near or in the molting period, as indicated by a soft carapace or incomplete exoskeleton hardening, were excluded from behavioral experiments.

Before the experiment, all crabs were temporarily housed in a recirculating aquaculture system (RAS) within the marine aquaculture laboratory of the institute. They were fed fresh frozen clam meat daily at 10:00. The water temperature was maintained at 22 °C, with a salinity range of 23–25, a pH of 7.7 ± 0.1, and dissolved oxygen levels between 5.5 and 6.1 mg·L^−1^.

### 2.2. Construction of the Behavior Observation System

**Figure 1:**
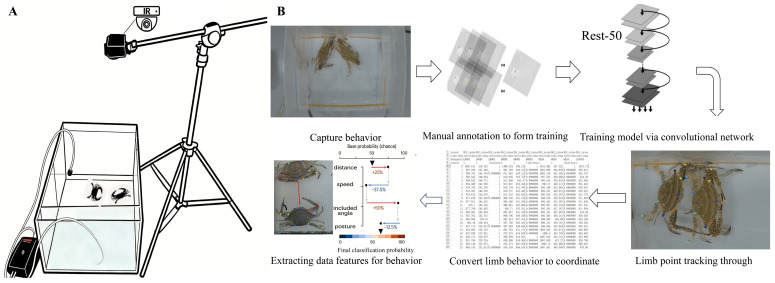
Experimental setup for behavioral quantification. (**A**) Schematic diagram of the resident–intruder arena, indicating the camera position and arena dimensions (50 cm × 35 cm × 25 cm). (**B**) Schematic diagram of the machine learning training pipeline, illustrating the workflow from video input and keypoint annotation to DeepLabCut pose estimation and subsequent Simple Behavioral Analysis (SimBA) behavioral classification into three categories: anxious behavior, demonstration displays, and fighting sequences.

The behavior monitoring system consisted of an experimental site, an air pump, vibration-damping foam pads, an elevated tripod, and a DTUP K9plus motion camera (Dongguan, China). The transparent acrylic arena (50 cm × 35 cm × 25 cm) featured two side partitions (each 5 cm from the side wall) equipped with air stones connected to the air pump, as shown in Figure 1A. Each partition wall had a set of 12 horizontally aligned perforations, each 10 mm in diameter, facilitating the exchange of oxygen-rich water between the aerated water zone and the experimental animal activity zone while minimizing bubble interference with video recording. To reduce vibration artifacts, the arena was placed on a 5 cm thick foam pad. The DTUP K9plus motion camera was mounted 50 cm above the center of the arena on the tripod, capturing all experimental footage at a 2K resolution (30 frames per second). Lighting conditions were kept constant throughout all recording sessions. Experiments were conducted in a room isolated from natural light, with LED ceiling lights positioned directly above the arena. The distance and angle between the light source and the arena were fixed across all trials to ensure consistent illumination.

### 2.3. Model Training for Postural Tracking and Behavioral Classification

In the present study, behavioral analysis was performed using DeepLabCut (version 3.0.0rc6), an open-source pose estimation tool. The software package was downloaded from the official GitHub repository (https://github.com/DeepLabCut/DeepLabCut, accessed on 10 July 2024). The implementation followed the methodology described by [32]. DeepLabCut is distributed under the GNU Lesser General Public License v3.0 (LGPL-3.0). Behavioral analysis was performed using SimBA, an open source tool distributed under a BSD 3-Clause License modified for academic and research use only. The software package was downloaded from the official GitHub repository (https://github.com/sronilsson/SimBA, accessed on 11 July 2024). Consistent with the license terms, the software was used exclusively for academic research purposes in this study.

DeepLabCut (DLC) and Simple Behavioral Analysis (SimBA) are implemented on a local server set up by the Institute of Oceanography at Ningbo University. They are executed via Python v. 3.6.13 and utilize a server equipped with an NVIDIA^®^ GeForce RTX™ 4070 Ti SUPER 24G GPU for model operations. The GUIs of DLC and SimBA are realized using XQuartz 11 and Tk v. 8.6.10.

This study establishes a deep learning-based pipeline for the quantitative analysis of crab behavior. Initially, we collected a total of 132 independent video clips that systematically document typical behavioral patterns exhibited by the crabs in experimental settings, including aggression, locomotion and threat displays, thereby ensuring the diversity and comprehensiveness of behavioral samples (Table 1). To accurately quantify these complex behaviors, we defined 13 key anatomical landmarks on the body surface of crabs, encompassing crucial regions such as the head, chelipeds, major joints, and swimming limbs (Table 2). As shown in Figure 1B, the spatial positions and dynamic changes in these landmarks allow for the complete reconstruction and characterization of the entire behavioral repertoire under investigation.

Regarding data processing, we selected 5280 representative image frames from the full set of video material, covering various behavioral phases, lighting conditions, and individual postures. All images were manually and meticulously annotated on a frame-by-frame basis by rigorously trained researchers to ensure accurate coordinate positions for all 13 key points in each image. This high-quality annotated dataset provided reliable supervision for subsequent model training. Based on this dataset, we developed a crab pose estimation model using the DeepLabCut framework with a ResNet-50 architecture. ResNet-50, a classic 50-layer deep residual network, incorporates skip connections that effectively mitigate the vanishing gradient problem in deep network training, rendering it highly effective for image feature extraction tasks [39]. Compared to deeper or shallower network variants, this architecture achieves an optimal balance between computational efficiency and model capacity, making it particularly suitable for training with limited annotated data while effectively avoiding overfitting risks, and has therefore been widely adopted in behavioral analysis research. During the model training phase, we performed a total of 500,000 optimization iterations. The annotated dataset of 5280 frames was randomly split into training (80%, 4224 frames) and testing (20%, 1056 frames) sets. Tracking accuracy was evaluated using the test set, with localization error defined as the Euclidean distance between predicted and manually annotated keypoint coordinates. The model achieved a mean test error of 5.66 ± 1.47 pixels, and a likelihood threshold of 0.95 was applied to filter low-confidence predictions. The training process involved feeding annotated images into the network, predicting keypoint coordinates via forward propagation, comparing these predictions with the ground truth annotations, and continuously adjusting the network weights using backpropagation to minimize prediction errors. This procedure enabled the model to progressively adapt from generic image recognition capabilities to the precise localization of specific anatomical structures in swimming crabs.

Based on established ethological criteria and preliminary observations, interactions were classified into three distinct categories: (1) demonstration displays (including chelae spreading and directed approaches), (2) anxious behaviors (encompassing vigilance and anxious pacing), and (3) fighting sequences (involving pushing, striking, mounting, and grappling).

Following the pose estimation workflow, the behavioral classification of the swimming crab was conducted using SimBA. The exported DeepLabCut CSV files, containing the (x, y) coordinate trajectories of all 13 anatomical landmarks across each frame of the 132 labeled video recordings, served as the input for SimBA’s multistage machine learning pipeline.

In accordance with the established protocols recommended by the SimBA developers, an ensemble Random Forest (RF) classifier was first constructed. The model integrated predictions from 100 individual decision trees (configured via the “RF n estimators” parameter). Each tree functioned as a flowchart that partitioned frames into behavioral categories based on kinematic features derived from the landmark coordinates [40]. During tree construction, the “auto RF max features” parameter governed the number of pose features evaluated at each split, while the “gini impurity criterion” was employed to assess the quality of proposed partitions at node divisions. To evaluate generalization performance, 20% of the annotated behavioral sequences (designated by the “test size” parameter) were withheld as an independent validation set. Tree growth was terminated when further splits did not improve separation, with a “sample leaf parameter” requiring a minimum of 1000 frames per terminal node.

Using this RF architecture, a specialized classifier was trained to detect aggressive displays, a key behavior of interest. The model learned discriminative spatiotemporal patterns from manually annotated grooming sequences, distinguishing them from non-aggressive behaviors such as locomotion and resting. Detection thresholds were optimized through researcher-led evaluation against ground truth video evidence, resulting in a confidence threshold of >0.5 and a minimum behavior duration of 180 ms for positive classification.

Once trained, the classifier was deployed to automatically identify and timestamp all instances of aggressive behavior across the full set of unlabeled videos. The output included detailed CSV files listing the start frame, end frame, duration, and confidence score for each detected bout, thereby enabling subsequent quantitative and statistical analysis (Figure 1B).

### 2.4. Quantitative Assessment and Verification of Aggressiveness in Swimming Crab

This study aims to establish a reliable quantitative analysis method for assessing aggressive behavior in swimming crab and to explore the reliability and practical value of the quantitative results. The experiment is divided into two main parts.

The first part focuses on developing a standardized attack test followed by neurochemical analysis. A novel paradigm of repetitive agonistic interaction was employed to accurately measure the TAI of individual crabs. Subsequently, neurobiological experiments were conducted to examine the potential correlation between the aggression index and specific neurotransmitter levels.

The second part applies the attack quantification method to obtain aggression indices for all individuals, stratifies the population into distinct aggression phenotypes based on these values, and conducts cross-group interaction experiments to evaluate behavioral consistency, population-level aggression distribution, and implications for commercial aquaculture.

#### 2.4.1. Experimental Design of Resistance Between Residents and Invaders

In order to objectively evaluate the aggressiveness, we used an improved resident intruder paradigm to conduct behavioral experiments [41]. First, healthy male crabs with fixed specifications were randomly assigned identification numbers (No. 1–200) and paired as No. 1–2, No. 3–4, …, No. 199–200. For each pair, the roles of resident and intruder were randomly assigned to ensure that the identity of the resident was not systematically biased by any intrinsic factor (e.g., size, weight, or capture time). This randomization was intended to simulate the unpredictable nature of territorial intrusions in natural settings, where an individual may encounter an intruder without prior advantage. The sample size was determined based on two considerations. First, comparable sample sizes have been used in crustacean behavior studies: Liang et al. [8] used approximately 125 individuals per developmental stage of swimming crab, and Sun et al. [35] used 240 male Chinese mitten crabs. Our study focuses on a single developmental stage, and a sample size of 200 provides adequate statistical power to detect individual variation in aggressiveness. Second, the sample size was also constrained by practical feasibility and animal availability, as wild-captured crabs meeting the inclusion criteria were limited. The chosen size of 200 balances statistical power with experimental feasibility. We then conducted behavioral experiments and recorded data for each confrontation group in turn.

Before the test, the resident crab was acclimatized in a standardized experimental site (60 cm × 40 cm × 30 cm, seawater from daily aquaculture water, with sufficient dissolved oxygen and appropriate temperature) for 10 min to establish its territorial dominance. Then the intruder crab was introduced and temporarily separated by a transparent acrylic partition to prevent immediate physical contact between the two crabs. After a 10 min adaptation period, the partition was removed and recording of all social interactions between the two crabs began within 30 min. Each group was repeated five times, with an interval of more than 24 h. All tests were carried out under controlled environmental conditions. During the experiment, we avoided any external interference. At the same time, constant water temperature and fixed light were maintained to eliminate confounding variables as far as possible. Finally, the high-definition videos were recorded and obtained.

The trained machine learning model was used to track the appendage postures of the high-definition experimental videos. At the same time, the behavior classification analysis was carried out, and the distribution of three different intensity attack behaviors during the experiment was accurately counted. The mean value of repeated results was taken as the final result.

#### 2.4.2. Group Classification and Cross-Validation Based on Aggressiveness

Quantitative aggression scores measured by the Time-Weighted Aggression Index (TAI) in the resident intruder experiment followed a normal distribution (Shapiro–Wilk test, *p*-value > 0.05). Considering the actual distribution, individuals with scores in the top 25% were classified as the high-aggression group (HA), and those in the bottom 25% as the low-aggression group (LA). The remaining 50% of the individuals were assigned to the moderate aggression group (MA). Furthermore, in order to evaluate the reliability of this classification, a series of binary interaction experiments was carried out by randomly selecting individuals from each classification group and setting them into homogeneous pairing (LA vs. LA and HA vs. HA, MA vs. MA) and heterogeneous pairing (LA vs. HA).

The experimental process refers to the above resident intruder antagonistic test design. Each test lasts 30 min, with special emphasis on counting the number of high-intensity aggressive behaviors that can cause direct physical injury (such as direct clamp, grappling, etc.). By comparing the interaction patterns between these groups, we evaluate the stability of attack ranking and clarify the potential impact of attack distribution on group dynamics.

### 2.5. Sample Collection and Neurotransmitter Quantification

A standardized protocol was employed to collect physiological samples and measure resting state hemolymph 5-HT levels in the experimental swimming crab. To minimize the influence of stress responses from behavioral experiments on physiological parameters, a 3-day recovery period was implemented following behavioral trials. Hemolymph samples (1 mL) were aseptically collected from the base of the crab appendages using a sterile syringe. The samples were immediately transferred to prechilled centrifuge tubes and processed at 4 °C (8000 r/min, 20 min) to separate cellular components. The resulting supernatant was aliquoted and stored at –80 °C. Concurrently, neural tissues (eyestalks) and peripheral tissues (muscle, hepatopancreas, gill, and heart) were dissected, rapidly frozen in liquid nitrogen, and maintained at –80 °C for subsequent analysis.

The concentrations of neurotransmitters were measured utilizing crab-specific ELISA kits (Sumai Biotechnology Technology, Yancheng, China) in accordance with the double antibody sandwich procedure from the manufacturer. All assays included standard curves and blank controls to ensure measurement accuracy. Special care was taken to maintain consistent low-temperature conditions throughout the procedures to prevent neurotransmitter degradation.

### 2.6. Statistical Analysis

Statistical analysis was conducted using SPSS 26.0 and Origin 2024. Behavioral data were expressed as mean values. Prior to analysis, Levene‘s test was employed to assess the homogeneity of variance, and the Kolmogorov–Smirnov test was used to examine the normality of data distribution. For data following a normal distribution, Pearson’s correlation analysis was performed, and differences in aggressive behavior between categorical groups were evaluated using independent samples *t*-tests. In cases where data deviated from normality even after transformation, Spearman’s correlation analysis was applied, and the Kruskal–Wallis test was used to compare differences between groups. To ensure comparability specifically for cross-method comparisons, data were standardized using z-score normalization only in this section of the analysis. A significance threshold of *p*-value < 0.05 was adopted for all statistical tests.

## 3. Results

### 3.1. Establishing an Integrated DeepLabCut-SimBA Pipeline for Behavioral Pose Estimation in Swimming Crab

**Figure 2:**
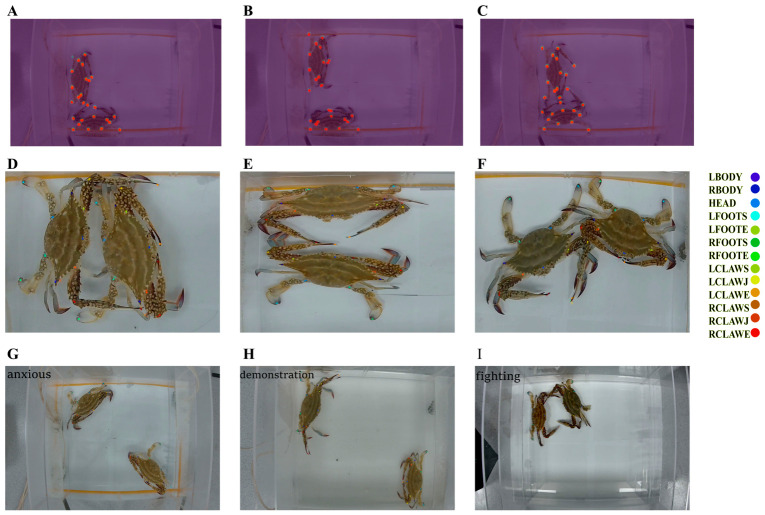
Representative outputs of the DeepLabCut-SimBA framework for pose tracking and behavioral classification in swimming crabs. (**A**–**C**) Tracking performance under challenging lighting conditions, demonstrating stable keypoint detection across multiple postures despite suboptimal illumination. (**D**–**F**) Tracking performance across different social interaction postures, showing that the model reliably tracks appendage keypoints during pushing, striking, and grappling behaviors. (**G**–**I**) Behavioral classification results, illustrating clear discrimination among the three supervised target behaviors: anxious behavior (**G**), demonstration displays (**H**), and fighting sequences (**I**).

**Figure 3:**
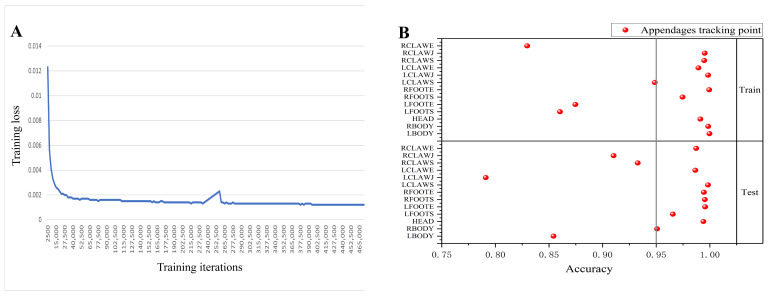
Performance assessment of the DeepLabCut-based keypoint tracking model for crab appendages. (**A**) Training loss curve showing a decrease from approximately 0.012 to below 0.002 over 500,000 iterations, indicating successful model convergence. (**B**) Tracking accuracy on the training and test sets, demonstrating that the model achieves reliable performance on both datasets.

To evaluate the robustness and generalizability of the pose estimation framework, we implemented a tiered validation strategy across progressively more challenging datasets. Representative tracking outputs are visualized in Figure 2. Initial model performance was assessed using the DeepLabCut “Evaluate Network” function on a held-out subset of frames derived from the training distribution. The final pose estimation model converged with a training loss below 0.002, as shown in Figure 3A. Localization error, defined as the average Euclidean distance (in pixels) between predicted coordinates and manual annotations, was employed as the primary accuracy metric. The model achieved a mean error of 3.87 ± 1.39 pixels on the training set and 5.66 ± 1.47 pixels on the test set, indicating strong predictive concordance with human annotators and confirming high localization fidelity under controlled conditions.

Building on this foundation, we further extended the validation process to evaluate the robustness of the model across large-scale, ecologically heterogeneous datasets. An independent validation set consisting of 1.62 million randomly sampled frames was constructed to assess the stability of predictions across a wide range of postures, lighting conditions, and behavioral contexts. By quantifying the agreement between predicted and ground truth coordinates across all 13 anatomical keypoints, we obtained a mean prediction accuracy of 95% (as shown in Figure 3B), underscoring the model’s capacity for reliable inference under variable recording conditions.

To transition from pose estimation to behavioral classification, we next conducted a systematic comparison between automated classifier outputs and manual annotations across the full test set. Per-class accuracy was 91% for anxious behaviors, 94% for demonstration displays, and 93% for fighting sequences, corresponding to a mean accuracy of 92.7% across the three behavioral categories. The overall misclassification rate among the three categories was 4.3%, with the remaining 3% of instances classified as unclassified (i.e., cases for which the model failed to assign a valid behavioral label). Collectively, these results demonstrate discriminative ability with limited confusion between behaviorally similar classes and a low rate of unclassified instances, supporting the applicability of the classifier for automated, high-throughput behavioral phenotyping in swimming crab.

### 3.2. Establishment of the Time-Weighted Aggression Index (TAI) Model

To quantitatively assess aggression in the swimming crab, this study developed a composite aggression index (TAI). We calculated aggression frequency for each individual as the total number of aggressive acts divided by the trial duration ($T_{\mathrm{total}}$ = 1800 s). This frequency served as the dependent variable in a multiple linear regression, with the durations of anxious behavior ($D_{\mathrm{anxious}}$), demonstration ($D_{\mathrm{demonstration}}$), fighting ($D_{\mathrm{fighting}}$), and the time of first attack ($T_{\mathrm{first}}$) as predictors. The regression coefficients were normalized and used as weights. The TAI formula is:

$$\mathrm{TAI}=\;((0.14\;\times \;D_{\mathrm{anxious}}+\;0.21\;\times \;D_{\mathrm{demonstration}}+\;0.35\;\times \;D_{\mathrm{fighting}})/T_{\mathrm{total}})\;+\;0.3\;\times \;((T_{\mathrm{total}}\;-\;T_{\mathrm{first}})/T_{\mathrm{total}})$$

The TAI is scaled to a 0–1 range, with higher scores indicating greater aggressiveness. This weighting scheme ensures that TAI captures both when the first attack occurs and how much time the crab spends on aggressive behaviors across the whole trial, including anxious behavior, demonstration, and fighting.

### 3.3. Validation of Model Quantification Results

**Figure 4:**
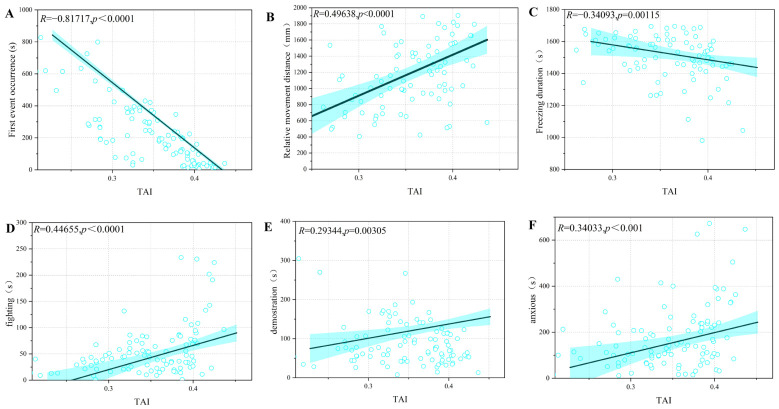
Correlation analysis between the Time-weighted Aggression Index (TAI) and six behavioral parameters. (**A**) First attack occurrence versus TAI (*R* = −0.817, *p*-value < 0.01). (**B**) Relative movement distance versus TAI (*R* = 0.496, *p*-value < 0.01). (**C**) Freezing duration versus TAI (*R* = −0.341, *p*-value < 0.01). (**D**) Fighting duration versus TAI (*R* = 0.447, *p*-value < 0.01). (**E**) Demonstration duration versus TAI (*R* = 0.293, *p*-value < 0.01). (**F**) Anxious behavior duration versus TAI (*R* = 0.340, *p*-value < 0.01). Each data point represents an individual behavioral observation. Solid lines indicate fitted regression lines for significant correlations (*p* < 0.05). The blue shaded area represents the 95% confidence interval.

To investigate the relationship between aggressive performance and TAI quantified scores in swimming crab, we quantified six behavioral indicators across 500 fighting trials (Figure 4). All six behavioral measures showed significant correlations with the aggression index (*p*-value < 0.01 in all cases). First event occurrence had the strongest correlation, negative as expected (*R* = −0.817). Relative movement distance came next, positively correlated with TAI (*R* = 0.496), followed by fighting duration (*R* = 0.447). Anxious behavior and demonstration showed weaker positive correlations (*R* = 0.340 and *R* = 0.293, respectively), while freezing duration was negatively correlated with TAI (*R* = −0.341). These results indicate that the aggression index captures key dimensions of aggressive behavior in swimming crab, with higher aggression scores corresponding to shorter initiation latencies, increased fighting duration, greater relative movement distance, and higher frequencies of anxious and demonstration behaviors, as well as reduced freezing time.

**Figure 5:**
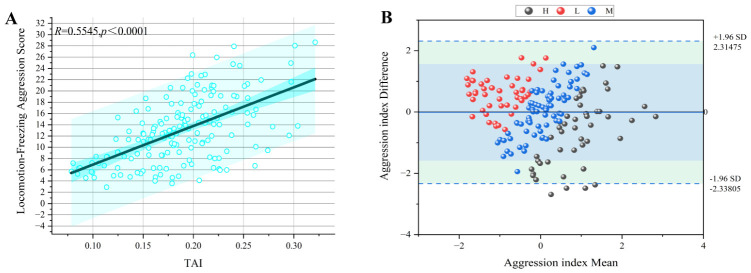
Comparison between the Time-weighted Aggression Index (TAI) and a previously established multiple linear regression model for aggression quantification. (**A**) Pearson correlation between TAI and the regression model predictions (*R* = 0.5545, *p*-value < 0.001, *n* = 200). The narrow dark blue shaded area around the regression line represents the 95% confidence interval of the regression line, and the wider light blue shaded area represents the 95% prediction interval for individual observations. (**B**) Bland–Altman plot showing the agreement between the two methods. The solid line indicates the mean difference, and dashed lines represent the 95% limits of agreement. The light green shaded area represents the 95% limits of agreement (+1.96 SD to −1.96 SD), and the inner light blue shaded area represents the 95% confidence interval of the mean difference. Nearly all differences fell within the limits, indicating no systematic bias between the two methods.

To further validate the quantitative aggression index, we compared the TAI against outputs from a previously established multiple linear regression model that predicts aggression based on relative movement distance ($X_{1}$) and freezing duration ($X_{2}$): “$Y\;=\;0.023X_{1}\;-\;0.001X_{2}\;-\;0.002\;(R^{2}\;=\;0.72)$” [8]. To make the comparison fair, we standardized both datasets using Z-score normalization. The Pearson correlation between our TAI and the predictions from this regression model was *R* = 0.555 (*p*-value < 0.001, Figure 5A), and the intraclass correlation coefficient (ICC(C,K)) was 0.713 (95%confidence interval (CI): 0.621–0.783, Table 3), indicating moderate agreement according to established criteria (ICC < 0.5 = poor, 0.5–0.75 = moderate, 0.75–0.9 = good, >0.9 = excellent) [42]. The moderate strength of these correlations is expected, given that the two methods approach aggression from fundamentally different angles. The regression model relies on two specific behavioral indicators (movement and freezing), whereas our TAI integrates multiple behavioral components (anxious behavior, demonstration, fighting, and attack latency). In other words, they capture related but distinct aspects of the overall aggressive phenotype. A Bland–Altman analysis further confirmed the agreement between the two methods, with nearly all differences falling within the 95% limits of agreement (Figure 5B), indicating no systematic bias. Taking these results together, the TAI shows good concordance with an established, behavior-based regression model. More importantly, beyond this statistical agreement, our method offers several practical advantages. The TAI is derived directly from video tracking, enabling high throughput and automated quantification. It is scaled to a 0–1 range, making it easy to interpret and compare across studies. Its composite formula is transparent and modular, and it can be readily adapted to other crustacean species or behavioral contexts by adjusting the component behaviors. These features make the TAI not just an alternative, but a practical and extensible tool for quantifying aggression in crustacean research.

### 3.4. Cluster Analysis of Aggression Expression and Serum 5-HT Levels

**Figure 6:**
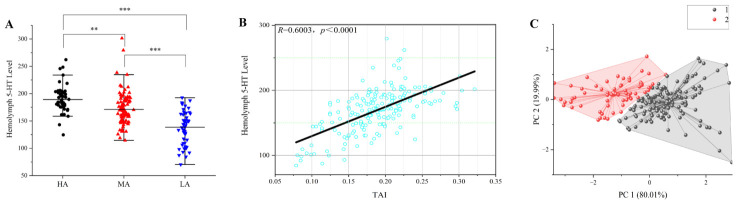
Association between serotonin (5-HT) levels and TAI. (**A**) Distribution of 5-HT levels among groups categorized by aggression intensity. (**B**) Correlation analysis between the aggression index and 5-HT levels. (**C**) K-means clustering of TAI and 5-HT. Large cluster (*n* = 160, 80.1%) showed expected positive relationship; small cluster (*n* = 40, 19.9%) deviated (high 5-HT, low aggression). The grey shaded area represents the large cluster, and the red shaded area represents the small cluster. Silhouette coefficient (0.52) indicated reasonable cluster quality, and bootstrap Jaccard similarities (>0.75) confirmed cluster stability. Asterisks indicate the significance of differences between the high- and low-aggression groups, with “**”, and “***” denoting significance levels of *p*-value < 0.01 and *p*-value < 0.001, respectively.

As illustrated in Figure 6A, hemolymph 5-HT levels exhibited significant variation among aggression intensity groups, and the overall distribution pattern closely mirrored the grouping defined by aggression index values, suggesting a consistent alignment between behavioral phenotyping and physiological profiling.

Combining individual Aggression Index scores with resting state hemolymph serotonin (5-HT) concentrations and grouping data based on aggression intensity, correlation and cluster analyses were performed. The results revealed a positive correlation between 5-HT levels and aggression (*R* = 0.6003, *p*-value < 0.001, Figure 6B). K-means cluster analysis further resolved the sample into two distinct 5-HT response subtypes (Figure 6C). One cluster (large, 80.1%) showed the expected positive correspondence between high 5-HT levels and high aggression scores. The second cluster (small, 19.9%) deviated from this pattern, with individuals exhibiting relatively high 5-HT levels but low aggression scores. Cluster quality and stability were confirmed by the silhouette coefficient (0.52) and bootstrap Jaccard similarities (>0.75). These findings are consistent with established evidence implicating serotonin as a key modulator of aggression in crustaceans [43], while also underscoring the complexity of 5-HT-mediated behavioral regulation. Collectively, these results demonstrate the utility of the TAI as a robust tool for probing physiologically relevant behavioral variation, reinforcing its value in studies of the neurochemical underpinnings of aggression.

### 3.5. Classification Outcomes and Practical Applications

**Figure 7:**
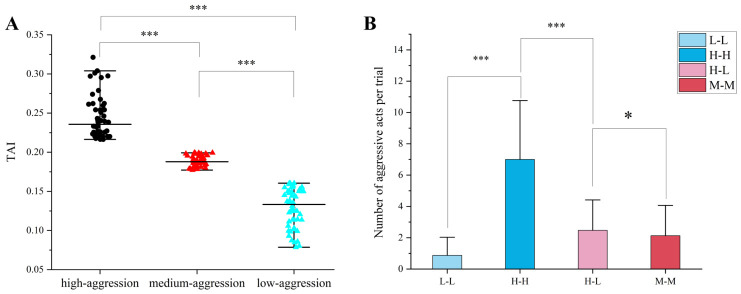
Validation of aggression index-based grouping. (**A**) Distribution of TAI scores across the three groups: high aggression (HA, top 25%), medium aggression (MA, middle 50%), and low aggression (LA, bottom 25%). Black dots, red triangles, and blue triangles represent HA, MA, and LA, respectively. (**B**) Comparison of aggressive acts per trial across different pairing contexts: H-H, M-M, L-L, and H-L. Asterisks indicate the significance of differences among groups, with “*” and “***” indicating that the difference significance level is 0.05 and 0.001, respectively.

According to the normal distribution characteristics of the aggregation index in the experimental population, individuals were classified into high-aggression (top 25%), low-aggression (bottom 25%), and medium-aggression (middle 50%) groups (Figure 7A). This 25% cut-off was chosen for two reasons. First, Fisher et al. [44] noted that including a wider range of scores in extreme-groups designs (e.g., 25% rather than 10%) substantially mitigates effect size inflation. Second, with a total sample size of 200, this cut-off yields 50 individuals per group, providing an adequate sample size for subsequent cross-validation. Additionally, as shown in Figure 7A, the middle 50% of individuals exhibited a pronounced clustering effect, making the quartile split a natural partition of the distribution. Random pairing experiments were then conducted for a total of 125 trials, with 25 trials each for L-L, H-H, and M-M pairings, and 50 trials for H-L pairings. The number of effective aggressive acts per trial was recorded. Systematic analysis across four pairing contexts (L-L, H-H, H-L, and M-M) revealed an interactive effect between intrinsic aggression traits and social context (Figure 7B). A Kruskal–Wallis test indicated significant differences among the four groups (H = 69.08, df = 3, *p*-value < 0.001).

Pairings between highly aggressive individuals (H-H) elicited the highest attack frequency (mean = 7.08 ± 3.66), significantly exceeding all other groups (Dunn’s post hoc, *p*-value < 0.001). In contrast, low-aggression pairings (L-L) showed the lowest attack frequency (mean = 0.96 ± 1.18), with 48% of trials recording zero attacks. Heterogeneous pairings (H-L) did not differ significantly from medium-aggression pairings (M-M) in attack frequency (means = 2.41 ± 1.97 and 2.25 ± 1.94, respectively; Bonferroni corrected *p*-value > 0.999).

## 4. Discussion

### 4.1. Integrating Computer Vision and Supervised Classification for High-Throughput Aggression Quantification in Swimming Crab

Conventional commercial tracking systems suffer from sensitivity limitations that hinder their flexible deployment in experiments involving diverse animal species [45]. Traditional ethological assessments of aggression in crustaceans, including the swimming crab, predominantly depend on manual observation and annotation of agonistic encounters, and such manual scoring is susceptible to low reliability among raters [46]. For example, aggression has been assessed by manually counting how many aggressive postures a crab displays during the mirror test [47], or by manually recording the types and frequencies of aggressive acts exhibited during dyadic fighting trials [48]. This methodology is intrinsically susceptible to observer-related inconsistencies, particularly during prolonged scoring sessions where the application of behavioral criteria may vary. Furthermore, the processing of nuanced, nonintuitive behavioral data demands extensive time and labor, imposing significant constraints on scalability, reproducibility, and the feasibility of large-scale, high-throughput phenotyping efforts.

Machine learning has gained widespread recognition in behavioral research as a powerful tool for analyzing complex animal behaviors. In the field of aquaculture, several artificial intelligence-based tools have been developed to analyze complex behavioral patterns, reflecting a progressive trajectory in the field. In recent years, a growing body of research has emerged. For example, researchers established a machine learning-based personality analysis method (PAML) to detect dynamic personality changes in the swimming crab under stress. [49]. Subsequently, a machine vision-based monitoring system was developed, integrating image segmentation and pose estimation algorithms to evaluate behaviors including reproduction, molting, and feeding [17]. The improved YOLOv5s model with Res2Net backbone and coordinate attention was applied to detect fish distribution and enable accurate bait feeding in fisheries [50]. More recently, DeepLabCut was employed to investigate behavioral alterations in mitten crab exposed to varying ammonia concentrations, demonstrating the utility of pose estimation tools for detecting environmental stressor effects [35]. Collectively, these studies underscore the growing role of machine learning in advancing behavioral research in crustacean species.

Building on this foundation, the development of robust and transferable analytical tools remains critical for facilitating high-throughput and standardized behavioral assessments. The SimBA model is assumed to have better transferability than other machine learning methods, as features are extracted from body point labeling systems, providing greater flexibility for behavior detection in different settings [40]. Integrated workflows employing DeepLabCut for pose estimation and SimBA for behavior classification have demonstrated utility in analyzing complex behaviors, such as defensive responses and grooming in rodents, showing strong concordance with manual scoring and proving suitable for high-throughput analysis [34]. In the present study, we present an integrated approach that combines computer vision with supervised behavioral classification to automate the extraction of aggression-related temporal metrics directly from video recordings. These metrics were then integrated into a quantification formula using weights derived from multiple linear regression, establishing a more efficient, standardized, objective, and easily implementable framework for assessing aggression across diverse experimental contexts. Nevertheless, the performance and generalizability of this framework may depend on several technical factors. First, the accuracy of pose estimation is contingent on dataset size and annotation quality; smaller or less diverse training sets may yield lower tracking fidelity. Second, the supervised classification approach relies on species-specific ethograms; adaptation to new species would necessitate the construction of customized behavior catalogs. Third, while our model performed well under our specific laboratory conditions, variations in camera setup, lighting, or water clarity could affect tracking accuracy. Acknowledging these dependencies, the open-source nature of DeepLabCut and SimBA facilitates such adaptations, and the modular design of TAI allows for straightforward modification of component behaviors and weighting schemes.

### 4.2. Behavioral and Neurochemical Validation of the Aggression Index

Understanding the physiological underpinnings of aggression has long been a central objective in behavioral research, as such knowledge is essential for developing effective intervention strategies. Pang et al. investigated the role of tryptophan hydroxylase (TPH) in L-tryptophan metabolism and its regulatory function in agonistic behavior of the Chinese mitten crab, elucidating the molecular mechanisms underlying aggression and providing a theoretical foundation for its artificial modulation in crustaceans [51]. Previous studies have established serotonin (5-HT) as a key modulator of aggression in crustaceans [27,29]. However, most of these investigations relied on coarse behavioral classifications, which may mask subtle links between physiology and behavior. To complement this mechanistic understanding, we quantified individual aggression levels in swimming crabs using a machine learning-based behavioral phenotyping approach. This allowed for a refined examination of the serotonin–aggression relationship. When combined with resting state hemolymph 5-HT measurements, the index revealed a positive correlation between 5-HT levels and aggression scores. Cluster analysis further separated the sample into two distinct 5-HT response subtypes, suggesting that the link between serotonin and aggression is not uniform. One cluster showed the expected positive relationship between high 5-HT concentrations and elevated aggression. The second cluster, however, deviated from this pattern, hinting at interactions between serotonin levels and other physiological or metabolic factors, such as receptor availability or additional neuromodulators. These findings align with existing evidence that serotonin is a key regulator of aggression in crustaceans, while also highlighting the complexity of serotonin-mediated behavioral control. More importantly, they demonstrate the utility of the TAI as a new method for capturing individual variation in aggression with continuous, high-resolution measurement. In doing so, the TAI can reveal previously obscured patterns in neurochemical behavioral relationships and offers a powerful tool for future studies on the multifactorial regulation of aggression.

Beyond its utility in probing neurochemical correlates, the aggression index also proved valuable for behavioral profiling and social interaction studies. This framework enabled the identification of individuals with markedly divergent aggression scores within the population, and subsequent fighting trials revealed significant differences in combat dynamics among individuals with distinct aggression scores. Validation via randomly paired antagonistic trials between differentially classified groups further demonstrated that while individual aggression traits serve as a primary predictor of aggressive behavior, they are significantly modulated by opponent characteristics. Homogeneous pairings maximized the expression of individual differences, whereas the “behavioral mitigation” observed in heterogeneous pairings likely stemmed from proactive avoidance strategies by less aggressive individuals, effectively reducing conflict opportunities even when the more aggressive individual retained a high attack propensity. These findings contribute to a more nuanced understanding of the adaptive regulation of aggression in animal social behavior, demonstrating that the actual occurrence of aggression is determined not only by intrinsic aggressiveness but also by the interactive behavioral strategies of both participants. Taken together, these results suggest that selective breeding of low-aggression lineages may represent a viable strategy for mitigating aggressive behavior in aquaculture production. As a concrete example of how the TAI can be applied in practice, consider its integration into selective breeding programs. Hatcheries could use the standardized resident-intruder paradigm described here to record fighting bouts of candidate broodstock and compute individual TAI scores. The lowest-scoring 25% of individuals, corresponding to the low-aggression group (LA) defined in this study, would then be selected as founders for low-aggression lineages. By repeating this selection process across generations, hatcheries could progressively reduce baseline aggressiveness in the population.

Collectively, these results support the utility of the TAI as a robust and versatile framework for quantifying individual variation in aggressive behavior. The index demonstrates strong predictive validity in both homogeneous and heterogeneous social contexts, captures physiologically relevant variation linked to serotonergic signaling, and reveals complexity in the serotonin–aggression relationship that may inform more targeted neurobiological inquiry. When integrated with selective breeding strategies, this approach holds promise for mitigating aggression-related losses in aquaculture, while also providing a scalable platform for basic research into the mechanisms underlying aggressive behavior in crustaceans.

### 4.3. Limitations of the Aggression Index Model

Several additional limitations should be acknowledged. One is that while our dataset of 5280 annotated frames was sufficient for model training and achieved satisfactory accuracy, larger and more diverse datasets (e.g., encompassing more individuals, postures, and lighting conditions) could further improve the robustness and generalizability of the pose estimation model. Another is that although all behavioral annotations were performed by rigorously trained researchers, manual labeling is inherently subject to inter-observer variability. We mitigated this by using consistent annotation protocols and cross-checking a subset of frames, but some degree of subjective bias may remain. A further limitation is that in the present study, the TAI framework and the trained classification model were applied to male swimming crab (*Portunus trituberculatus*) of a fixed-size class (60 ± 20 g) under controlled laboratory conditions. Therefore, direct application of the current model to other crustacean species, different size classes or developmental stages, or more naturalistic field settings would require species-specific ethogram adjustments and potentially full model retraining. With these considerations in mind, we now discuss the main limitations of the present study.

First, the experimental subjects were sampled exclusively from a single developmental stage of the swimming crab. Consequently, this design does not capture potential variations in the Time-weighted Aggression Index (TAI) across different ontogenetic phases. The absence of longitudinal or cross-developmental comparisons precludes the determination of whether the quantified aggressive behavioral tendencies remain stable throughout a longer life history trajectory or exhibit significant changes during maturation, molting, or senescence. Moreover, methodological constraints inherent to the underwater experimental setup must be acknowledged. Although carefully controlled, the measurements of aggressive encounters could be subtly influenced by optical distortion due to light refraction at the air-water interface. Furthermore, splashing and turbulence generated by the crabs’ vigorous movements during contests, while minimized, represent an unavoidable source of minor environmental noise in video recording and behavioral scoring.

In this study, the experiments were conducted under stable, controlled laboratory conditions. While this allows for high internal validity by isolating key variables, it necessarily simplifies the complex and dynamic milieu of natural habitats. The findings may not fully translate to wild settings where numerous uncontrolled biotic (e.g., predator presence, diverse competitors) and abiotic (e.g., fluctuating temperature, salinity, turbidity) factors could modulate the expression and outcome of aggressive interactions. Direct extrapolation to natural ecosystems should therefore be made with caution. Furthermore, the correlation analysis between aggression and hemolymph 5-hydroxytryptamine (5-HT) levels, while informative, is fundamentally correlational. The study did not investigate individual variation in the expression, distribution, or sensitivity of relevant 5-HT receptor subtypes within the neural circuitry governing aggression. This omission limits the depth of our conclusion’s mechanism, as the precise neurophysiological pathway linking serotonin to observed behavioral phenotypes still needs to be elucidated.

Despite these limitations, the experimental environment was deliberately designed to approximate conditions found in intensive aquaculture systems. Therefore, the findings regarding aggressive behavior and its physiological correlates retain significant practical relevance for crab husbandry and management under controlled rearing conditions, offering a valuable empirical reference for mitigating aggression-related losses in aquaculture practices.

## 5. Conclusions

Within the scope of the present study (male swimming crab *P*. *trituberculatus* of a fixed-size class under controlled laboratory conditions), this study applied an integrated machine learning pipeline combining DeepLabCut and SimBA to enable standardized classification of aggressive behavior in the swimming crab. By converting behavioral video data into quantifiable durations of aggression-related events, we established a Time-weighted Aggression Index (TAI) that allows high-throughput assessment of aggression. The TAI supports automated quantification and is straightforward to interpret for comparing individual differences in aggressiveness. Its formula is transparent and modular, and this modularity offers the potential for adaptation to other crustacean species or behavioral contexts. These features make the TAI a practical and extensible tool for quantifying aggression in male swimming crab under controlled conditions. This approach may help advance fundamental research on aggressive behavior and provide value for aquaculture management and selective breeding programs in crustacean species under controlled rearing settings.

## Figures and Tables

**Table 1:** Description of aggression-related behaviors in swimming crabs.

Behavior	Description
Directed approach	The crab moves steadily towards the target crab with continuous distance reduction, primarily using lateral or posterior forward locomotion to approach.
Circuitous approach	A general movement toward the target crab characterized by a meandering, back-and-forth trajectory, with the approaching crab adjusting its actions based on the target’s behavior.
Vigilance	The chelipeds are folded against the cephalothorax or exhibit minor opening/closing motions (<20°). The crab maintains a persistent orientation towards the tank corner with an outward burrowing tendency, often positioning its dorsal or lateralside toward the target.
Chelae spreading	Rapid, repetitive opening and closing of both chelipeds, with the maximum opening angle at the joint remaining within 90°.
Retreat	The experimental crab actively increases its distance from the target crab following an agonistic interaction.
Anxious pacing	After attempting to maximize distance from the target, the crab engages in sustained ambivalent locomotion, pacing repetitively along the edges of the experimental arena.
Pushing	The crab uses its chelipeds to push against the opponent while forcefully propelling itself forward with its walking appendages, attempting to displace the opponent through physical force.
Striking	The crab suddenly uses one or both chelipeds to deliver a rapid blow or strike to the opponent.
Grappling	The crab uses its major cheliped to grasp and pinch the opponent’s carapace, cheliped, or walking appendages.
Mounting	One crab climbs onto the dorsal carapace of the opponent, engaging in repeated mounting and dismounting movements.

Note: Based on the ethogram defined above, behavioral annotations were manually performed on 132 video clips. These annotations served as the ground truth for training the SimBA classifier.

**Table 2:** Key marker points on crab appendages.

Mark Point	Anatomical Position	Definition
LBODY	Left posterolateral carapace spine	The apex of the prominent posterolateral spine on the left side of the carapace.
RBOD	Right posterolateral carapace spine	The apex of the prominent posterolateral spine on the right side of the carapace.
HEAD	Anterior cephalothorax midline	The midpoint on the anterior margin of the carapace, between the bases of the paired antennules.
LFOOTS	Left 5th pereiopod coxal base	The proximal articulation (coxopodite) of the left 5th pereiopod (swimming appendages) with the cephalothorax.
LFOOTE	Left 5th pereiopod dactylus tip	The distal extremity of the dactylopodite on the left 5th pereiopod.
RFOOTS	Right 5th pereiopod coxal base	The proximal articulation (coxopodite) of the right 5th pereiopod (swimming appendages) with the cephalothorax.
RFOOTE	Right 5th pereiopod dactylus tip	The distal extremity of the dactylopodite on the right 5th pereiopod.
LCLAWS	Left cheliped (1st pereiopod) coxal base	The proximal coxal articulation of the left major cheliped (1st pereiopod).
LCLAWJ	Left cheliped carpus propodus joint	The primary flexion joint between the carpus and propodus of the left major cheliped.
LCLAWE	Left cheliped dactylus tip	The distal apex of the movable finger (dactylus) of the left major cheliped.
RCLAWS	Right cheliped (1st pereiopod) coxal base	The proximal coxal articulation of the right major cheliped (1st pereiopod).
RCLAWJ	Right cheliped carpus propodus joint	The primary flexion joint between the carpus and propodus of the right major cheliped.
RCLAWE	Right cheliped dactylus tip	The distal apex of the movable finger (dactylus) of the right major cheliped.

Note: These 13 anatomical keypoints were defined based on the morphological characteristics of *Portunus trituberculatus* and served as the positional reference for annotating the training dataset of the limb tracking model.

**Table 3:** Intraclass correlation coefficients between automated and manual behavioral quantification methods.

ICC Type	ICC Value	95% CI
ICC(C,1)	0.554	0.451–0.644
ICC(C,K)	0.713	0.621–0.783

Note: ICC(C,1) and ICC(C,K) denote intraclass correlation coefficients for single and average measurements, respectively, based on a two-way mixed-effects model with consistency agreement (*n* = 200 crabs). According to established criteria: ICC < 0.5 = poor, 0.5–0.75 = moderate, 0.75–0.9 = good, >0.9 = excellent. The ICC(C,K) value of 0.713 therefore indicates moderate agreement between the two methods.

## Data Availability

The raw data supporting the conclusions of this article will be made available by the authors upon reasonable request, without undue reservation.

## Abbreviations

The following abbreviations are used in this manuscript:

5-HT	Serotonin
DLC	DeepLabCut
DA	Dopamine
SimBA	Simple Behavioral Analysis
TAI	Time-weighted Aggression Index
